# Leptin and advanced glycation end products receptor (RAGE) in tuberculosis patients

**DOI:** 10.1371/journal.pone.0254198

**Published:** 2021-07-02

**Authors:** Tássia Kirchmann Lazzari, Erika Cavalheiro, Sandra Eugênia Coutinho, Lívia Fontes da Silva, Denise Rossato Silva

**Affiliations:** 1 Programa de Pós-Graduação em Ciências Pneumológicas, Universidade Federal do Rio Grande do Sul, Porto Alegre, Brazil; 2 Faculdade de Medicina, Universidade Federal do Rio Grande do Sul, Porto Alegre, Brazil; 3 Hospital de Clínicas de Porto Alegre, Porto Alegre, Brazil; Massachusetts General Hospital/Harvard Medical School, UNITED STATES

## Abstract

**Introduction:**

The pathogenesis of consumptive syndrome of tuberculosis (TB) is largely unknown. Leptin concentrations may be high because of the host’s inflammatory response, contributing to weight loss in patients with TB. The receptor for advanced glycation end products (RAGE) is also associated with weight loss in patients with TB and is related to enhanced mortality. The objective of this study was to evaluate the association between leptin and AGE/RAGE.

**Methods:**

Case-control study. Leptin, AGE (carboxymethyl lysine, CML) and soluble RAGE (sRAGE) were measured from blood samples by ELISA.

**Results:**

We included in the study 34 patients with TB and 34 controls. We found an inverse correlation between serum leptin levels and sRAGE, only in cases (r = -0.609, p < 0.0001). sRAGE levels were lower in patients with TB who died as compared with patients who survive (21.90 ± 4.24 pg/mL vs 66.14 ± 29.49 pg/mL; p = 0.045). Leptin levels were higher in patients with TB who died as compared with patients who survive (14.11 [7.48–14.11] ng/mL vs 3.08 [0.54–6.34] ng/mL; p = 0.028).

**Conclusions:**

We identified lower sRAGE levels and higher leptin levels in patients with TB who died as compared with patients who survive. In addition, an inverse and significant correlation between serum leptin and sRAGE levels was demonstrated. Future studies, with a larger sample size and in different settings, including not only hospitalized patients, are needed to confirm these findings.

## Introduction

Tuberculosis (TB) is an infectious disease with a high worldwide rate of morbidity and mortality. The consumptive nature of TB is related to anorexia and metabolic alterations, whose pathogenesis is largely unknown. Inflammatory cytokines are initially suggested as causative agents, but there is not enough evidence [1].

In the last decades, leptin emerged as a candidate for mediating metabolic changes related to the consumptive syndrome of TB. It may be involved in the interaction between immune response and the nutritional status in TB. Low leptin concentrations due to decreased body fat may be associated with decrease immunity and worse disease outcome. In addition, leptin concentrations may be high because of the host’s inflammatory response, suppressing appetite and contributing to weight loss in patients with TB [2–6].

The receptor for advanced glycation end products (RAGE) is also associated with weight loss in patients with TB [7]. AGEs are a diverse group of irreversible products resulting from glycation between reducing sugars and amino groups in proteins, lipids, and nucleic acids [8, 9]. RAGE is expressed in normal lungs, at low levels, and becomes upregulated during inflammation and infection [10–13]. One study demonstrated that RAGE is higher in patients with TB than in controls, and is related to enhanced mortality [7].

Both leptin and RAGE are associated with weight loss and worse outcomes, and there are no previous studies evaluating both in patients with TB. Therefore, the aim of this study was to evaluate the association between leptin and AGE/RAGE in patients with TB and controls.

## Methods

### Study design and location

We conducted a prospective case-control study in a tertiary care, university-affiliated hospital (Hospital de Clínicas de Porto Alegre–HCPA), from January 2017 to December 2019, to evaluate the association between leptin and AGE/RAGE in patients with TB and controls. The Ethics Committee at HCPA approved the study number 110580/140044), and all subjects gave written informed consent to participate. We hypothesize that there is a correlation between leptin and AGE/RAGE. Patients with TB were recruited at HCPA inpatient’s units, and the control group consisted of volunteers recruited in the same hospital, matched for sex and age in a 1:1 matching ratio. The control group consisted of volunteers recruited in the same hospital, selected among members of the patients’ family (who were accompanying the patients at the hospital). We decided this because cohabitants are exposed to the same risk factors for tuberculosis and family members who cohabit with patients with TB were examined to exclude active TB and to detect latent TB. If family member have active or latent TB, he/she was not included in the study. The only comorbidity allowed among the controls was HIV, because it is a comorbidity that is also prevalent among the cases.

### Patients and data collection

Patients with TB diagnosis, older than 18 years, who agreed to participate, were included in the study. Patients with extrapulmonary TB, and those who had been on pulmonary TB treatment for more than 3 days, and patients and controls with diabetes, previous TB and pregnant women were excluded from the study.

After signing informed written consent enrolled subjects were interviewed using a standardized questionnaire. Demographic and clinical data were recorded. The results of the main diagnostic tests performed were also recorded, as well as the outcome of hospitalization (discharge or death). The diagnosis of pulmonary TB was based on consensus criteria [14]. Sputum smears were stained by Ziehl-Neelsen (ZN) staining technique for the detection of acid-fast bacilli, and culture was performed using the Ogawa-Kudoh method. Drug sensitivity testing was performed. All patients and controls were tested for HIV.

### Laboratory tests

Blood sample was collected between 8 and 9 AM after an overnight fast, centrifuged and frozen at -80°C. Leptin, CML, and RAGE were measured by Elisa, according to the manufacturer’s instructions, and the quality control passed (leptin: Linco Research, St. Charles, MO—EZHL-80SK; CML: OxiSelect N-epsilon-(Carboxymethyl) Lysine [Cell Biolabs Inc, San Diego, CA]—CELL-STA816; RAGE: Human RAGE Quantikine [R&D Systems Inc., Minneapolis, MN]—RAB0007).

### Statistical analysis

Data analysis was performed using IBM SPSS Statistics for Windows, version 22.0 (Armonk, NY, IBM Corp). Data were presented as number of cases, mean ± standard deviation (SD), or median with interquartile range (IQR). Shapiro-Wilk test was used for testing normality. Categorical comparisons were carried out by chi-square test (McNemar). Continuous variables were compared using the paired *t*-test or Wilcoxon test. Pearson’s (or Spearman’s when indicated) correlations was performed to evaluate for potential relationships. Multivariable logistic regression analysis was performed to evaluate if the factors statistically significant in univariate analysis were independently associated with case/control status. The goodness of fit of the multiple logistic regression model was assessed using the Hosmer-Lemeshow test. Odds ratios (ORs) and nominal 95% confidence intervals (CIs) were presented. A two-sided p value ≤ 0.05 was considered significant for all analyses. The sample size was calculated based on the expected correlation between sRAGE and leptin levels of 0.50. Considering a confidence level of 95% and a power of 80%, we estimated a sample of at least 29 individuals per group.

## Results

**Fig 1:**
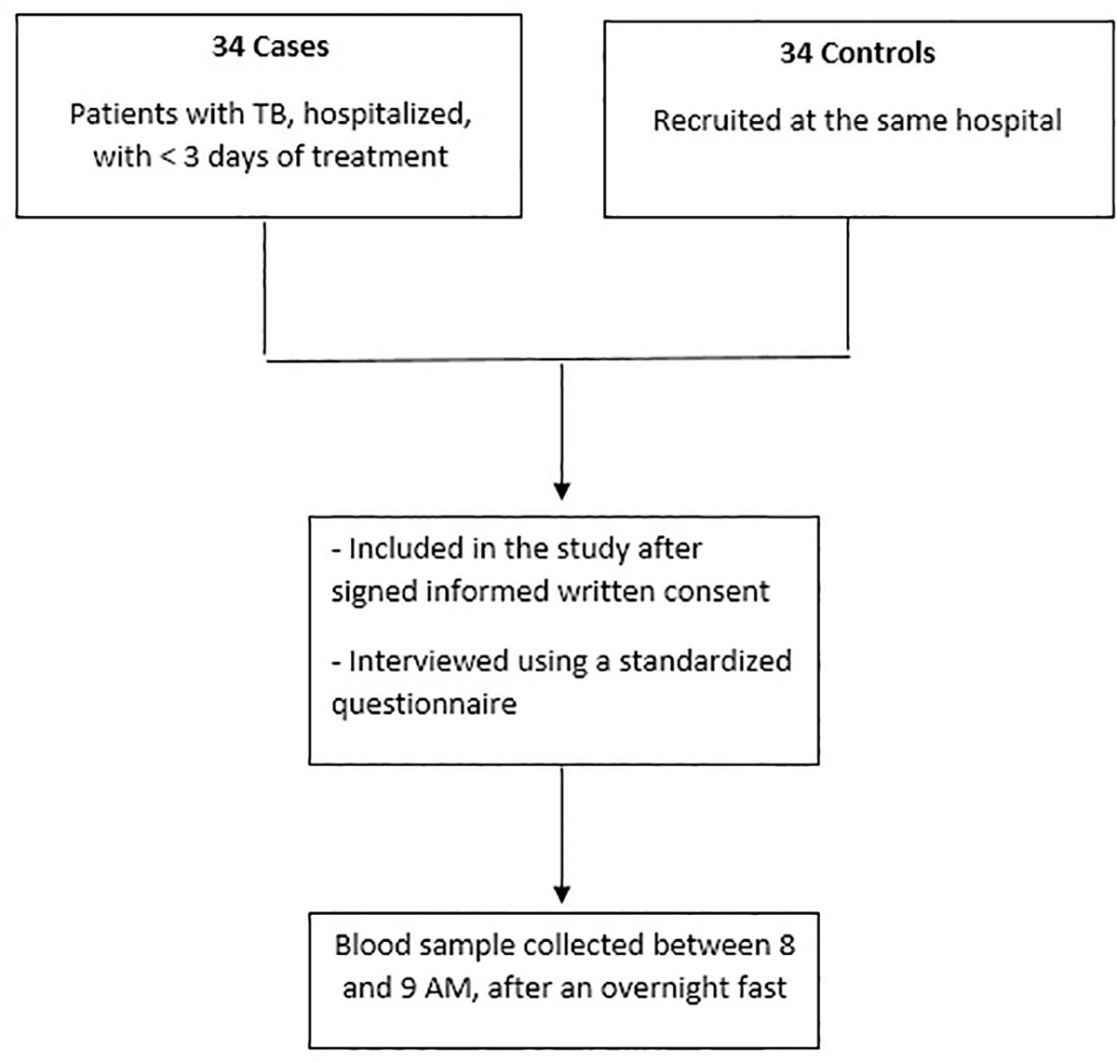
Flow chart of patients included in the study.

During the study period, 34 patients with TB and 34 controls met the inclusion criteria and were included in the analysis (Fig 1). Table 1 shows the comparison between cases and controls. White race was more common among controls than in cases (82.4% vs 52.9%, p = 0.01). HIV positivity was more frequent in cases as compared with controls (41.2 vs 8.8, p = 0.002), and was the only comorbidity present in cases and controls. TB cases had a lower BMI than controls (19.6 ± 3.6 vs 27.0 ± 5.1, p<0.0001). There was no statistically significant difference in CML, sRAGE and leptin levels between cases and controls.

**Table 1:** Comparison between cases and controls.

Variables	Cases n = 34	Controls n = 34	p value
Male sex, n (%)	23 (67.6)	23 (67.6)	-
Age (years), mean ± SD	36.7 ± 16.3	37.7 ± 15.7	0.786
White race, n (%)	18 (52.9)	28 (82.4)	0.01
Current smoking, n (%)	21 (61.8)	15 (44.1)	0.145
Alcohol abuse, n (%)	13 (38.2)	6 (17.6)	0.059
Drug use, n (%)	13 (38.2)	7 (20.6)	0.110
HIV positive, n (%)	14 (41.2)	3 (8.8)	0.002
BMI (kg/m^2^), mean ± SD	19.6 ± 3.6	27.0 ± 5.1	<0.0001
Serum CML (μg/μL), median (IQR)	0.06 (0.03–0.10)	0.07 (0.03–0.14)	0.453
sRAGE (pg/mL), mean ± SD	63.5 ± 30.5	57.5 ± 24.0	0.366
Leptin (ng/mL), median (IQR)	3.30 (0.55–6.68)	4.41 (1.81–7.54)	0.105

SD: standard deviation. HIV: human immunodeficiency syndrome. BMI: body mass index. CML: carboxymethyl lysine. IQR: interquartile range. sRAGE: soluble receptor of advanced glycation end products.

Table 2 shows the correlations between leptin, CML, and sRAGE. We found an inverse correlation between serum leptin levels and sRAGE, only in cases (r = -0.609, p < 0.0001).

**Table 2:** Bivariate correlations with leptin, CML and sRAGE in patients with TB and controls.

	Cases		Controls	
	Leptin		Leptin	
	r	p Value	r	p Value
**CML**	0.265	0.129	-0.057	0.751
**sRAGE**	-0.609	<0.0001	-0.055	0.755

CML: carboxymethyl lysine. sRAGE: soluble receptor of advanced glycation end products.

In a multivariable analysis model including white race, HIV positivity, BMI, sRAGE, and leptin levels, only white race and BMI were independently associated with case status (Table 3).

**Table 3:** Multivariable analysis of factors associated with case status.

Characteristic	β	SE	Wald	OR (95% CI)	p Value
White race	2.618	1.061	6.091	0.073 (0.009–0.583)	0.014
HIV infection	-1.335	1.060	1.586	3.800 (0.476–30.345)	0.208
BMI	0.630	0.182	12.001	0.533 (0.373–0.761)	0.001
sRAGE	0.010	0.019	0.269	0.990 (0.953–1.028)	0.604
Leptin	-0.087	0.107	0.668	1.091 (0.885–1.345)	0.414

HIV: human immunodeficiency syndrome. BMI: body mass index. sRAGE: soluble receptor of advanced glycation end products.

Among TB cases, sRAGE, CML and leptin levels were not different between patients who lost weight and those who did not. sRAGE levels were lower in patients with TB who died as compared with patients who survive (21.90 ± 4.24 pg/mL vs 66.14 ± 29.49 pg/mL; p = 0.045). Leptin levels were higher in patients with TB who died as compared with patients who survive (14.11 [7.48–14.11] ng/mL vs 3.08 [0.54–6.34] ng/mL; p = 0.028). There was no difference in CML levels between survivors and non-survivors.

## Discussion

In this case-control study, we aimed to evaluate the association between leptin and AGE/RAGE in patients with TB and controls. We identified lower sRAGE levels in patients with TB who died as compared with patients who survive. Also, leptin levels were higher among patients with TB non-survivors than in survivors. In addition, we found an inverse and significant correlation between serum leptin and sRAGE levels, in TB cases.

RAGE is a receptor that is involved in pulmonary inflammation and infection. It is expressed at low levels in normal lung and is upregulated during conditions associated with inflammation, lung damage, and infection [10–13]. In the present study, we found lower sRAGE levels in patients with TB who died. In a study conducted in murine models, van Zoelen et al demonstrated that RAGE deficient mice displayed more body weight loss and enhanced mortality [15]. In a previous case-controls study [7], lower sRAGE levels were also associated with mortality.

Leptin is an adipokine initially implicated in the regulation of food intake and body weight [16]. In TB, due to decreased body fat, leptin concentrations may be low; however, high leptin levels were also described as a result of the host’s inflammatory response [3–6]. It has also an important role in the modulation of innate and adaptive immune responses, and the levels of expression of leptin in serum are increased after acute inflammation [16]. In our study, patients with TB non-survivors had higher leptin levels as compared with survivors. There are no previous studies demonstrating this association in patients with TB. However, elevated leptin levels have already been observed in association with mortality from other diseases [17, 18]. In an investigation with older adults, high leptin levels are predictive of cardiovascular mortality [18]. High leptin levels nearly doubled overall mortality in males [17].

We demonstrated, in patients with TB, an inverse and significant correlation between serum leptin and sRAGE levels. This association between leptin and RAGE was previously demonstrated in a type 2 diabetes mouse model. In this study, the authors showed that the leptin activity can inhibit RAGE expression in β-cells [19].

This study has some methodological limitations. First, the investigation was done in a single center, and the results may thus not apply to other settings. Second, only hospitalized patients were included in the study, probably more severe TB cases. Last, the small sample size precluded us to conduct a multivariable analysis of factors associated with mortality in patients with TB. In addition, white race and lower BMI were more frequent among patients with TB; although these are characteristics know to be associated with the disease, it would be important that, in future studies, the cases and controls be paired for these variables as well. In spite of these concerns, the strength of the present study is that it is the first study to demonstrate the correlation between serum leptin and sRAGE levels in patients with TB, and also the first one to show higher leptin levels in patients with TB who died. Nevertheless, our study was much more hypothesis-generating than designed to answer key mechanistic questions on the roles of RAGE/leptin in tuberculosis and the use of sRAGE/leptin as a reliable prognostic tool in tuberculosis.

In conclusion, in this study we identified lower sRAGE levels and higher leptin levels in patients with TB who died as compared with patients who survive. In addition, an inverse and significant correlation between serum leptin and sRAGE levels was demonstrated. Future studies, with a larger sample size and in different settings, including not only hospitalized patients, are needed to confirm these findings.

## References

[1] Schwenk A, Macallan DC. Tuberculosis, malnutrition and wasting. Current Opinion in Clinical Nutrition and Metabolic Care. Curr Opin Clin Nutr Metab Care. 2000;3: 285–291. doi: 10.1097/00075197-200007000-00008 10929675

[2] Skupien EC, Lazzari TK, Coutinho SE, Silva DR. The relation between leptin and inflammatory markers with respiratory and peripheral muscle strength in tuberculosis: A case–control study. Clin Respir J. 2018;12: 2559–2565. doi: 10.1111/crj.12956 30180300

[3] Schwenk A, Hodgson L, Rayner CFJ, Griffin GE, Macallan DC. Leptin and energy metabolism in pulmonary tuberculosis. Am J Clin Nutr. 2003;77: 392–398. doi: 10.1093/ajcn/77.2.392 12540399

[4] Van Crevel R, Karyadi E, Netea MG, Verhoef H, Nelwan RHH, West CE, et al. Decreased plasma leptin concentrations in tuberculosis patients are associated with wasting and inflammation. J Clin Endocrinol Metab. 2002;87: 758–763. doi: 10.1210/jcem.87.2.8228 11836317

[5] Buyukoglan H, Gulmez I, Kelestimur F, Kart L, Oymak FS, Demir R, et al. Leptin levels in various manifestations of pulmonary tuberculosis. Mediators Inflamm. 2007;2007:64859. doi: 10.1155/2007/64859 17497033 PMC1804295

[6] Yüksel I, Şencan M, Dökmetaş HS, Dökmetaş I, Ataseven H, Yönem Ö. The relation between serum leptin levels and body fat mass in patients with active lung tuberculosis. Endocr Res. 2003;29: 257–264. doi: 10.1081/erc-120025033 14535627

[7] Da Silva LF, Skupien EC, Lazzari TK, Holler SR, De Almeida EGC, Zampieri LR, et al. Advanced glycation end products (AGE) and receptor for AGE (RAGE) in patients with active tuberculosis, and their relationship between food intake and nutritional status. PLoS One. 2019;14:e0213991. doi: 10.1371/journal.pone.0213991 30870511 PMC6417785

[8] Goldin A, Beckman JA, Schmidt AM, Creager MA. Advanced Glycation End Products: Sparking the Development of Diabetic Vascular Injury. Circulation. 2006;114: 597–605. doi: 10.1161/CIRCULATIONAHA.106.621854 16894049

[9] Singh R, Barden A, Mori T, Beilin L. Advanced glycation end-products: A review. Diabetologia. 2001;44:129–146. doi: 10.1007/s001250051591 11270668

[10] Wittkowski H, Sturrock A, Van Zoelen MAD, Viemann D, Van Der Poll T, Hoidal JR, et al. Neutrophil-derived S100A12 in acute lung injury and respiratory distress syndrome. Crit Care Med. 2007;35: 1369–1375. doi: 10.1097/01.CCM.0000262386.32287.29 17414728

[11] Uchida T, Shirasawa M, Ware LB, Kojima K, Hata Y, Makita K, et al. Receptor for advanced glycation end-products is a marker of type I cell injury in acute lung injury. Am J Respir Crit Care Med. 2006;173: 1008–1015. doi: 10.1164/rccm.200509-1477OC 16456142 PMC2662912

[12] Morbini P, Villa C, Campo I, Zorzetto M, Inghilleri S, Luisetti M. The receptor for advanced glycation end products and its ligands: A new inflammatory pathway in lung disease? Mod Pathol. 2006;19: 1437–1445. doi: 10.1038/modpathol.3800661 16941014

[13] Cheng C, Tsuneyama K, Kominami R, Shinohara H, Sakurai S, Yonekura H, et al. Expression profiling of endogenous secretory receptor for advanced glycation end products in human organs. Mod Pathol. 2005;18: 1385–1396. doi: 10.1038/modpathol.3800450 15933755

[14] Conde MB, Melo FAF de, Marques AMC, Cardoso NC, Pinheiro VGF, Dalcin P de TR, et al. III Brazilian Thoracic Association Guidelines on tuberculosis. J Bras Pneumol. 2009;35: 1018–48. doi: 10.1590/s1806-37132009001000011 19918635

[15] van Zoelen MAD, Wieland CW, van der Windt GJW, Florquin S, Nawroth PP, Bierhaus A, et al. Receptor for advanced glycation end products is protective during murine tuberculosis. Mol Immunol. 2012;52: 183–189. doi: 10.1016/j.molimm.2012.05.014 22698798

[16] Iikuni N, Kwan Lam Q, Lu L, Matarese G, Cava A. Leptin and Inflammation. Curr Immunol Rev. 2008;4: 70–79. doi: 10.2174/157339508784325046 20198122 PMC2829991

[17] Amrock SM, Weitzman M. Effect of increased leptin and C-reactive protein levels on mortality: Results from the national health and nutrition examination survey. Atherosclerosis. 2014;236: 1–6. doi: 10.1016/j.atherosclerosis.2014.06.009 24998933 PMC4399234

[18] Batsis JA, Sahakyan KR, Singh P, Bartels SJ, Somers VK, Lopez-Jimenez F. Leptin, adiposity, and mortality: Results from the national health and nutrition examination survey III, 1988 to 1994. Mayo Clin Proc. 2015;90: 481–491. doi: 10.1016/j.mayocp.2015.01.023 25841252

[19] Han D, Yamamoto Y, Munesue S, Motoyoshi S, Saito H, Win MTT, et al. Induction of receptor for advanced glycation end products by insufficient leptin action triggers pancreatic β-cell failure in type 2 diabetes. Genes to Cells. 2013;18: 302–314. doi: 10.1111/gtc.12036 23410183

